# Editorial: Immunoelectron microscopy: Placing molecular functions within a neuronal context

**DOI:** 10.3389/fnana.2022.1043371

**Published:** 2022-10-24

**Authors:** Rafael Luján, María Eulalia Rubio

**Affiliations:** ^1^Synaptic Structure Laboratory, Departamento de Ciencias Médicas, Instituto de Investigación en Discapacidades Neurológicas, Facultad de Medicina, Universidad Castilla-La Mancha, Campus Biosanitario, Albacete, Spain; ^2^Department of Neurobiology, University of Pittsburgh School of Medicine, Pittsburgh, PA, United States; ^3^Department of Otolaryngology, University of Pittsburgh School of Medicine, Pittsburgh, PA, United States

**Keywords:** electron microscopy, pre-embedding immunogold, post-embedding immunogold, FIB/SEM, freeze-fracture

The anatomical demonstration of the synapse, a concept proposed by Santiago Ramón y Cajal but first named by Sherrington, became a reality in the 1950s, thanks to electron microscopists (Robertson, 1953; Gray, 1959). During the last 60 years, electron microscopy has continuously been an essential tool to investigate the complex structures of nerve cells and their connections with other cells. However, to unravel many neuronal processes implicated in physiological and pathological conditions, ultrastructural studies must be complemented with data regarding the subcellular localization of many specific molecules subserving a wide range of functions in the brain. Such data is provided by immunoelectron microscopy (immuno-EM). Thus, combining the spatial resolution of electron microscopy, the ability of antibodies to bind proteins specifically, and quantitative approaches, immuno-EM has revolutionized ultrastructural investigations of the molecular structure of synapses and neurites within the brain, becoming an essential tool to link the information obtained by biochemistry, cell biology, and electrophysiology.

Unraveling the molecular architecture of neurons and neural circuits remains one of neuroscience's significant challenges. For such challenges, immuno-EM techniques allow the identification and localization of the molecules involved in brain processes, which is one of the first steps toward understanding how neural circuits contribute to the functional organization of the nervous system, both in health and disease. In addition, immuno-EM allows quantitative morphometric analysis, allowing us to detect normal and abnormal molecular and structural alterations within brain cells.

In this Frontiers Research Topic entitled “*Immunoelectron microscopy: Placing molecular functions within a neuronal context*,” we aim to provide an updated and practical overview of different methods of immuno-EM. We also intended to emphasize the great potentiality of immunolabeling as an indispensable tool that contributes to the understanding of structural-functional relationships. We have combined original research and review articles by pioneering researchers in the field covering conventional and newly developed immuno-EM methodologies. In this collection, Egaña-Huguet et al. use pre-embedding double labeling HRP/gold techniques to determine the effect of the constitutive deletion of the TRPV1 gene on the expression and localization of some elements of the endocannabinoid system in the rodent hippocampus. Ulloa-Navas et al. use surgical resections from focal cortical dysplasia patients with pre-embedding immunogold to identify and classify subpopulations of oligodendrocytes within the white matter of human cortices. Roldán-Sastre et al. combine quantitative pre-embedding and post-embedding immunogold to determine the cellular and subcellular localization of Gαo, a member of the pertussis toxin-sensitive Gi/o family and a major signal transducer of specific GPCRs, in the cerebellar cortex. Petralia and Wang provide a well-needed review of the post-embedding immunogold technique. They comprehensively compare the immunolabeling protocol used in their laboratory with earlier and more recent methods used by other laboratories around the World. Luján et al. present a pioneering study that combines the FIB/SEM technology with pre-embedding immunogold and newly developed 3D quantitative tools to show that SK2 channel density differs between excitatory and inhibitory neurons and demonstrates a large variability in the density of SK2 channels in spines. Eguchi et al. summarize morphological parameters of the CaV_2_ distribution obtained using sodium dodecyl sulfate-digested freeze-fracture replica labeling (SDS-FRL). They emphasize that such distribution is synapse type-specific and could contribute to functional properties in synaptic transmission. Turegano-Lopez et al. present an effective method for the 3D reconstruction of labeled subcellular structures at the ultrastructural level after single-neuron labeling in fixed tissue. With this study, the authors show that this procedure can help unravel the connectivity between neural elements at different scales, which is highly needed considering the high complexity of the nervous tissue. Guerrero-Given et al. present a new software package that automatically and precisely determines the number, density, distribution, and clustering arrangement of cellular proteins on immunogold-labeled SDS-FRL replicas. The precise quantification of proteins at a specific neural state can be used to gain insight into cellular function.

Together, this Research Topic brings the readers conventional and recently developed applications of quantitative immunoelectron microscopical techniques. The papers in this Research Topic show that immuno-EM is an objective approach that escapes any conjecture as to where molecules localize, thus banishing the idea that immunoelectron microscopy is a purely morphological and “descriptive” approach. Our goal with this Research Topic is to offer new perspectives on molecular, structural, and functional associations of neurons and glial cells in the brain at the ultrastructural level. We hope that the research papers will help and guide senior and junior neuroscientists to define strategies that need to be considered when exploring the organization of molecules in health and disease.

## Author contributions

All authors listed have made a substantial, direct, and intellectual contribution to the work and approved it for publication.

## Funding

This work was supported by grants from the Spanish Ministerio de Economía y Competitividad (RTI2018-095812-B-I00 and PID2020-118511RB-I00) and Junta de Comunidades de Castilla-La Mancha (SBPLY/17/180501/000229 and SBPLY/21/180501/000064) to RL and the National Institutes of Health (DC013048) to MER.

## Conflict of interest

The authors declare that the research was conducted in the absence of any commercial or financial relationships that could be construed as a potential conflict of interest.

## Publisher's note

All claims expressed in this article are solely those of the authors and do not necessarily represent those of their affiliated organizations, or those of the publisher, the editors and the reviewers. Any product that may be evaluated in this article, or claim that may be made by its manufacturer, is not guaranteed or endorsed by the publisher.

## References

[B1] Gray E. G. (1959). Electron microscopy of synaptic contacts on dendrite spines of the cerebral cortex. Nature 183, 1592–1593. 10.1038/1831592a0 13666826

[B2] Robertson J. D. (1953). Ultrastructure of two invertebrate synapses. Proc. Soc. Exp. Biol. Med. 82, 219–223. 10.3181/00379727-82-20071 13037850

